# The molecular dimension of microbial species: 2. *Synechococcus* strains representative of putative ecotypes inhabiting different depths in the Mushroom Spring microbial mat exhibit different adaptive and acclimative responses to light

**DOI:** 10.3389/fmicb.2015.00626

**Published:** 2015-06-29

**Authors:** Shane Nowack, Millie T. Olsen, George A. Schaible, Eric D. Becraft, Gaozhong Shen, Isaac Klapper, Donald A. Bryant, David M. Ward

**Affiliations:** ^1^Department of Mathematical Sciences, Montana State University, BozemanMT, USA; ^2^School of Environmental Sciences, University of Guelph, GuelphON, Canada; ^3^Department of Land Resources and Environmental Sciences, Montana State University, BozemanMT, USA; ^4^Department of Biochemistry and Molecular Biology, The Pennsylvania State University, University ParkPA, USA; ^5^Department of Mathematics, Temple University, Philadelphia, PAUSA; ^6^Department of Chemistry and Biochemistry, Montana State University, BozemanMT, USA

**Keywords:** microbial species, light adaptation, light acclimation, cyanobacteria, photosynthesis

## Abstract

Closely related strains of thermophilic *Synechococcus* were cultivated from the microbial mats found in the eﬄuent channels of Mushroom Spring, Yellowstone National Park (YNP). These strains have identical or nearly identical 16S rRNA sequences but are representative of separate, predicted putative ecotype (PE) populations, which were identified by using the more highly resolving *psaA* locus and which predominate at different vertical positions within the 1-mm-thick upper-green layer of the mat. Pyrosequencing confirmed that each strain contained a single, predominant *psaA* genotype. Strains differed in growth rate as a function of irradiance. A strain with a *psaA* genotype corresponding to a predicted PE that predominates near the mat surface grew fastest at high irradiances, whereas strains with *psaA* genotypes representative of predominant subsurface populations grew faster at low irradiance and exhibited greater sensitivity to abrupt shifts to high light. The high-light-adapted and low-light-adapted strains also exhibited differences in pigment content and the composition of the photosynthetic apparatus (photosystem ratio) when grown under different light intensities. Cells representative of the different strains had similar morphologies under low-light conditions, but under high-light conditions, cells of low-light-adapted strains became elongated and formed short chains of cells. Collectively, the results presented here are consistent with the hypothesis that closely related, but distinct, ecological species of *Synechococcus* occupy different light niches in the Mushroom Spring microbial mat and acclimate differently to changing light environments.

## Introduction

As reviewed by the first paper in this three-paper series on the molecular dimension of microbial species (Becraft et al., 2015), in the course of a long-term effort to understand the composition, structure, and function of microbial mat communities inhabiting alkaline, siliceous hot springs in Yellowstone National Park (YNP), we have focused on sequence variation in increasingly more divergent genes, progressing from 16S rRNA (Ferris et al., 1997) to the 16S–23S rRNA internal transcribed spacer region (Ferris et al., 2003) to protein-encoding genes (Becraft et al., 2011; Melendrez et al., 2011). Due to different cyanobacterial gene sequence variants being predominant at different locations along thermal gradients, we hypothesized the existence of temperature-adapted *Synechococcus* ecotypes, which was later demonstrated by obtaining representative strains and studying their temperature preferences (Allewalt et al., 2006). Similar temperature adaptations were reported for *Synechococcus* strains cultivated from Oregon hot springs by Peary and Castenholz (1964) and Miller and Castenholz (2000).

Differences in the distribution of 16S rRNA and 16S–23S internal-transcribed-spacer sequence variants along vertical profiles in the upper 1 mm-thick photic zone of these mats (Ramsing et al., 2000; Ferris et al., 2003) led us to hypothesize the existence of different light-adapted *Synechococcus* ecotypes. Microsensor studies have shown that the dense populations of mat inhabitants alter light quantity and wavelength distribution dramatically with depth in the upper 1–2 mm of the mat (see Figure 4 in Becraft et al., 2015), providing selection conditions for evolutionary adaptations along these light gradients and to other environmental parameters that vary with depth. Additionally, microsensor analyses revealed that oxygenic photosynthesis in a mat recovering from physical disturbance exhibited two maxima, one nearer, and another farther from the mat surface (Ferris et al., 1997), providing further evidence in support of the existence of *Synechococcus* ecotypes adapted to different light microenvironments. Becraft et al. (2015) combined pyrosequencing analysis of the gene encoding *psaA*, a core subunit of the photosystem I reaction center, with analysis of the *psaA* sequences by Ecotype Simulation, an algorithm based on the Stable Ecotype Model of species and speciation that predicts ecological species populations from sequence variation (Koeppel et al., 2008). These hypothetical species are called putative ecotypes (PEs) until they are shown to exhibit properties expected of ecological species (Becraft et al., 2015). This analysis permitted prediction of *Synechococcus* PEs and provided a conceptual basis for ensuing studies of their vertical distributions in the microbial mats. By examining 80 μm-thick vertical sections of mat samples collected at 60–63°C, which were obtained by cryotome sectioning, a progression from the mat surface downward of *Synechococcus* PEs B′9, A1, A4, A14, and A6 was observed (**Table 1**; see also Figures 3 and 4 in Becraft et al., 2015). The predicted A-like PEs exhibit identical or nearly identical 16S rRNA sequences (see Olsen et al., 2015).

**Table 1:** Summary of *psaA* Ti454-barcode sequencing analyses of *Synechococcus* strains.

				Dominant variant sequences^c^			Other sequences^e^	
Strain name	Temperature^a^	Depth^b^	Total sequences	Putative ecotype (PE)^d^	DVs	%	Total	%
JA-3-3Ab	58–65°C	400–600	1116	All	931	83.4		
				**A1**	930	83.3	185	16.6
				A14	1	0.1		
JA-2-3B′a (2–13)	51–61°C	N/A	2054	All	1690	82.3		
				**B′19**	1385	67.4	364	17.8
				B′24	300	14.6		
				B′11	5	0.2		
CIW-10	51–61°C	N/A	1528	All	1077	70.5		
				**B′24**	438	28.7	413	27
				€	639	41.8	38	2.5
65AY6Li	65°C	400–600	980	**A1**	780	79.6	200	20.4
65AY6A5	65°C	640–720	1325	All	1106	83.5	219	16.5
				**A4**	1105	83.4		
				A1	1	0.1		
60AY4M2	60°C	800	920	**A14**	756	82.2	164	17.8

*^a^Temperature of the sample from which the strain was obtained.*

*^b^Depth from surface where PE is most abundant at 60°C in Mushroom Spring microbial mat. Units are μm. N/A means PE was not a dominant population in the 60°C mat at any depth. Data reported in Becraft et al. (2015).*

*^c^Number of sequences that are identical to the dominant variant (DV; identical sequences that made up the plurality of a putative ecotype PE clade) of the PE listed in the same row.*

*^d^PEs in bold are the dominant PEs in the culture.*

*^e^Other sequences that are closely related to the DV sequence.*

*^€^A dominant sequence variant in the culture that is distinct from PE B′24 by 17 single-nucleotide polymorphisms.*

The existence of ecotypes of another hot spring cyanobacterium, *Plectonema notatum*, that are evolutionarily adapted to different irradiance levels, was demonstrated by Sheridan (1976, 1978). These findings, however, contrast with previous studies of light responses of native *Synechococcus* populations in such mats (Brock and Brock, 1969; Madigan and Brock, 1977), which had been interpreted as acclimative changes of a single *Synechococcus* population that was physiologically adjusting to a change in the environment (M.T. Madigan, personal communication). [Note: We will use the term acclimation to mean the physiological response of an organism to an environmental change; we will use adaptation to mean an alteration in the structure or function of an organism or any of its parts that results *from natural selection* and by which the organism becomes better fitted to survive and multiply in its environment.] If evolutionarily adapted *Synechococcus* ecotypes exist, changes in the relative abundances of differently adapted *Synechococcus* ecotypes, such as those observed in light alteration experiments by Becraft et al. (2015), would provide an alternate explanation of the responses observed in earlier studies.

Previous studies of evolutionary adaptation to light were performed on *Synechococcus* strains obtained from low-dilution enrichments with 16S rRNA sequences representative of predominant natural populations (Allewalt et al., 2006) or substrains therefrom (Kilian et al., 2007). As such, these strains might contain multiple ecotypes with the same 16S rRNA sequence, which were shown to exist by higher-molecular resolution, theory-based analyses (Becraft et al., 2011, 2015). Here we report on *Synechococcus* strains obtained from more highly diluted mat samples, which are representative of PEs designated from *psaA* sequence variation. These *Synechococcus* cultures are not axenic because they presently contain heterotrophic contaminants, but we refer to them as strains since we will show that they represent individual genotypes of distinct *Synechococcus* PEs (i.e., with respect to the cultivated *Synechococcus*, they are strains of a known species). We will demonstrate that strains representative of PEs that are known to predominate at different positions along the vertical light gradient in the mat have different evolutionary adaptations and acclimative responses to light, even though they have identical or nearly identical 16S rRNA sequences. The genomic sequences of these strains are described in the third paper of this series (Olsen et al., 2015).

## Materials and Methods

### Sample Collection

Samples for cultivation were collected from Mushroom Spring, YNP, at sites with temperatures of 60, 63, and 65°C on September 7, 2010 using a #4 cork borer (8 mm diameter). The top green layer, ∼1 mm in thickness, of each mat sample was removed with a razor blade, placed in a 1.5-mL microcentrifuge tube, and then returned to the lab in a thermos containing Mushroom Spring source water (cooled to the temperature at which the sample was collected). The time from sampling to the lab was ∼2 h, and the temperature in the thermos was ∼7 to 10°C degrees cooler upon arrival at the lab than that measured at the field site.

### Microscope Counts

To obtain an estimate of the number of *Synechococcus* cells found in the top green layer of a #4 mat core, the green layer of a 63°C mat sample was homogenized in 10 mL of autoclaved Mushroom Spring water. The cells in a 10-μL subsample were counted using a Bright-line hemacytometer (Hausser Scientific, Horsham, PA, USA) and microscope (Zeiss Axioskop 2 plus with an HBO 100 UV lamp) at 40× magnification. *Synechococcus* cells were identified by their orange–red autofluorescence.

### *Synechococcus* Isolation

Using Castenholz’s (1969) medium D supplemented with HEPES buffer (DH), Allewalt et al. (2006) were only successful in cultivating *Synechococcus* strains out to 10^5^-fold dilution of mat samples that contained ∼10^8^ to 10^9^ *Synechococcus* cells/mL. For this study, we developed a modified medium containing sodium acetate and yeast extract (medium DHAY), each added at 0.01% (w/v), which resulted in the recovery of strains of *Synechococcus* from more highly diluted mat samples and faster growth rates in liquid cultures. The medium was solidified with 1.5% (w/v) Gelrite^TM^ (Sigma–Aldrich, St. Louis, MO, USA) throughout the isolation process.

Mat samples containing *Synechococcus* cells were diluted 10-fold to extinction prior to inoculation into autoclaved media that had been cooled to 50°C, which was then poured into sterile 60 mm-diameter Petri dishes. After allowing the Gelrite to solidify, the plates were placed in sealed Ziploc bags with wetted paper towels and were then incubated at 52°C under 50 μmol photons m^-2^sec^-1^ of white fluorescent light. Plates were inspected daily over a one-month period to monitor the growth of *Synechococcus* colonies. Isolated colonies formed by *Synechococcus* that grew on plates inoculated with mat samples that were diluted at least 10^5^-fold from the original mat sample were picked with a sterile toothpick. In order to focus efforts on strains representative of different PEs, we screened strains by *psaA* sequence analysis before attempting further purification. Thus, to obtain sufficient material for sequencing, colonies were suspended in 2 mL of liquid medium DHAY in glass test tubes with caps that were loosely fastened to allow gas exchange. The strains were grown to stationary phase and were then scaled up in a step-wise manner to 20 mL and then to 100 mL by the addition of DHAY medium. Incubation conditions were as described above and remained constant throughout this process.

### Molecular Assessment of *psaA* Genotype, Strain Purification, and Purity

#### DNA Extraction and PCR Amplification

Cells from a 1.5-mL aliquot of liquid culture were pelleted by centrifugation for 3 min at 4800 × *g* and resuspended in 200 μL of lysis buffer. Cells were lysed using the FastPrep Cell Disrupter (Bio101 Savant Instruments, Holbrook, NY, USA) and DNA was extracted and purified using the FastDNA Spin kit (Molecular Biosciences, Boulder, CO, USA) by following the manufacturer’s instructions. Amplicons of *psaA* for DNA sequencing were produced by polymerase chain reaction (PCR) amplification as described by Becraft et al. (2011).

#### Sanger Sequencing and Ecotype Assignment

Sanger sequencing of *psaA* amplicons was performed at the Idaho State University Molecular Biosciences Core Facility. *Synechococcus* strain sequences were aligned to sequences obtained from the mat, which were used to demarcate PEs (Becraft et al., 2015). Batch cultures producing amplicons with no ambiguous base calls and low background signals, and with sequences that were identical to a dominant variant (DV) representative of a predominant PE, were further purified.

#### Purification of Strains

Strains representative of different *psaA*-based PEs were diluted to extinction again in liquid medium DHAY to increase the probability that strains were representative of a single PE. The resulting highest-dilution subculture was prepared for pyrosequencing (see methods below). Strains with sequences containing ambiguous base calls and/or high background signal were re-diluted to extinction on plates, and the cultivation process was repeated by picking well-isolated colonies from high-dilution plates.

#### Pyrosequencing for Assessing Culture Purity

Strains meeting the purity criteria described above were grown to late exponential growth phase (between 2 and 5 × 10^7^ cells/mL), and the cells from 60-mL liquid cultures were pelleted by centrifugation for 30 min at 1000 × *g*. Cell pellets were flash-frozen in liquid nitrogen and stored at -80°C. In order to have DNA of sufficient quality for both assessing culture purity and genome sequencing (see Olsen et al., 2015), an enzymatic cell lysis protocol was followed. Each frozen pellet was thawed and resuspended in medium DHAY to produce a 1-mL total volume; DNA was extracted by a phenol/chloroform/isoamyl alcohol method following cell lysis by lysozyme/proteinase K (see complete DNA extraction protocol in Supplementary information). RNA and other impurities were removed using the RNase I treatment protocol described by the Department of Energy Joint Genome Institute^1^. The DNA concentration was quantified using a NanoDrop Spectrophotometer ND-1000 (NanoDrop Technologies, Wilmington, DE, USA), and PCR was performed as described above to ensure that the DNA could be amplified.

As was the case in Allewalt et al. (2006), cyanobacterial strains contained heterotrophic contaminants, which we could not eliminate with repeated streaking for isolation or using spent media from the mixed strains or pure-cultures of heterotrophic isolates. To characterize the composition of the strains, pyrosequencing of 16S rRNA amplicons produced with primers 28F and 519R was performed according to the methodology posted on The Research and Testing Laboratory website^2^. Pyrosequencing of *psaA* gene amplicons was performed as described in Becraft et al. (2015) to assess the complexity of the cultivated *Synechococcus* population. Systematic errors, defined as single-nucleotide polymorphisms that were common to different strains, were removed. A culture was considered to contain a single predominant cyanobacterial ecotype if it contained a single dominant *psaA* sequence, as well as closely related, less-abundant genetic variants with 1 or 2 randomly distributed nucleotide substitutions compared to the dominant sequence. The genetic variants might have arisen during cultivation or represent sequencing error (Gilles et al., 2011), which might have occurred because a non-high-fidelity polymerase (Qiagen HotStar Taq polymerase) was used. These sequences are available upon request.

#### Final Strain Purification and Purity Check

To achieve an extra level of confidence regarding purity with respect to cyanobacterial PEs, all strains were again diluted to extinction in liquid medium DHAY and the subculture from the highest dilution was subjected to a second round of *psaA* amplicon sequencing using pyrosequencing. This sequence-based criterion was also used to test the purity of the two *Synechococcus* strains (JA-3-3Ab and JA-2-3B′a (2–13)) that were originally cultivated by Allewalt et al. (2006), and whose genomes were sequenced (Bhaya et al., 2007), and a third *Synechococcus* strain (CIW-10), which was derived from strain JA-2-3B′a (2–13) by Kilian et al. (2007; see Results).

### Growth at Different Irradiances

All growth experiments were performed with medium DHAY using an illuminated growth chamber that consisted of an aquarium of dimensions 152.4 cm × 15.2 cm × 38.1 cm that served as a water bath. The temperature (52 or 60°C) was controlled with a PolyScience circulator, Series 7000 (Niles, IL, USA). The chamber was illuminated with two identical ATI 6 × 80 W SunPower T5 high-output fluorescent fixtures (Denver, CO, USA), one on each side of the aquarium, using a total of 12 150-cm-long fluorescent tubes of type F80W-T5-841-ECO (General Electric, Fairfield, CN, USA). Different light conditions, spanning the range of irradiances observed in nature, were achieved by applying various layers of neutral-density filter covering (GAM products, Los Angeles, CA, USA) around the culture tubes. Before sterilizing the culture tubes, irradiance was measured using a scalar irradiance probe, model QSL2100 from Biospherical Instruments (San Diego, CA, USA), by submersing the probe in 100 mL of water. To reduce the chance of CO_2_ limitation, a sterilized, cotton-plugged 7 mm glass tube was connected to a gas cylinder containing 6% CO_2_ in air (GENDCO, Bozeman, MT, USA) and was used to sparge the cultures at approximately one bubble per second. To prevent possible acidification of the medium, 26 mM NaHCO_3_ was added to the growth medium (before autoclaving). The optimal concentration of NaHCO_3_ was determined in a preliminary experiment by sparging (∼1 bubble/second) liquid medium DHAY with 6% v/v CO_2_ and identifying the minimum concentration of NaHCO_3_ required to stabilize the pH over a period of 3 days.

Prior to inoculation, liquid cultures were pre-grown to late exponential phase (to minimize lag phase), or to a density of ∼2 × 10^7^ cells/mL, at either (i) 52°C and at a scalar irradiance of 50 μmol photons m^-2^sec^-1^, or (ii) 60°C and 500 μmol photons m^-2^sec^-1^ of white fluorescent light under a supply of 6% CO_2_ in air, as described above. Duplicate cultures for each light intensity were inoculated by adding either 5.5 × 10^5^ cells/mL or 1 × 10^6^ cells/mL to 100 mL of medium DHAY in 175-mL glass P/T culture tubes (Bellco, Vineland, NJ, USA). The tubes were capped with silicon sponge closures (Sigma–Aldrich, St. Louis, MO, USA) to allow for gas exchange. Light intensity and pH were measured before and after each experiment to ensure that these parameters had remained stable during the experiment. Samples (1.0 mL) were taken every 12 h, fixed with glutaraldehyde (0.125% final concentration), and frozen at -80°C. Cell counts were obtained using flow cytometry (BD-FACSCanto or BD-FACSAria II flow cytometer and BD counting beads (BD Biosciences, San Jose, CA, USA) or CountBright Absolute counting beads (Invitrogen, Life Technologies, Waltham, MA, USA)). Each sample was filtered through a 70-μm screen-cap filter (Fisher Scientific) before analysis on the cell counter to avoid clogging of the flow cell. Growth rates were determined by estimating log-linear slopes during exponential growth phase.

**FIGURE 1:**
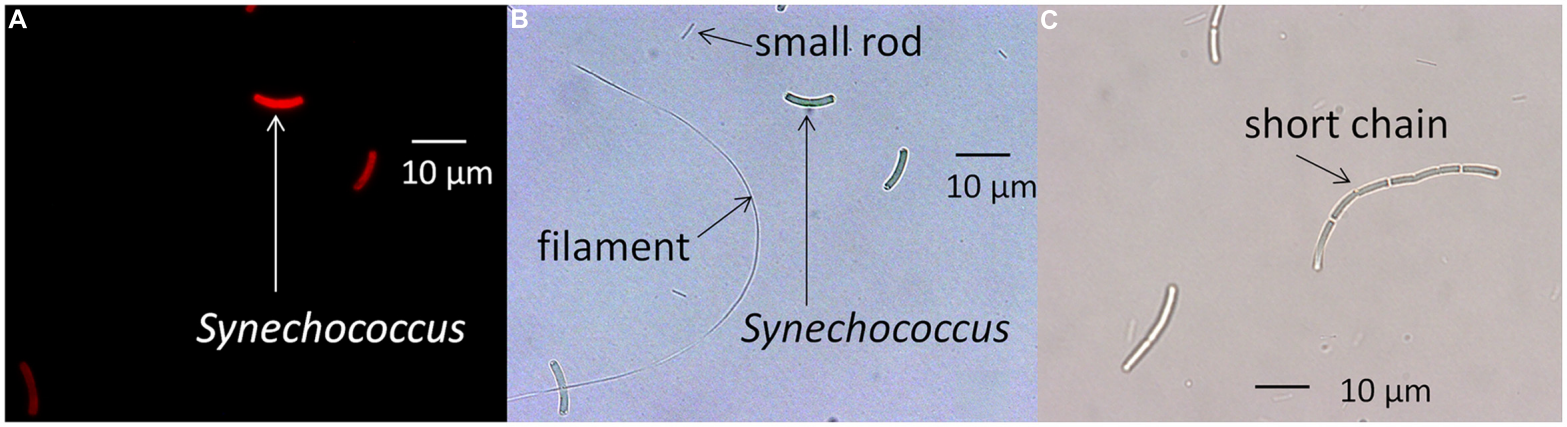
**Microscopic images of a *Synechococcus* strain and heterotrophic contaminants. (A)** Fluorescence microscopy photomicrograph of the PE A1 strain (65AY6Li) grown at a scalar irradiance of 50 μmol photons m^-2^sec^-1^. **(B)** Same image using differential interference contrast microscopy, showing rod-shaped and filamentous heterotrophic contaminants. **(C)** Differential interference contrast photomicrograph showing elongated cells of the PE A14 strain grown at a scalar irradiance of 600 μmol photons m^-2^sec^-1^ after pre-growth at 50 μmol photons m^-2^sec^-1^. Scale bars are 10 μm.

*Synechococcus* cells are typically 8–10 μm in length, and microscopic examination revealed that they were noticeably longer than any of the heterotrophic cells that were detected in the culture and that were also able to pass through the screen-cap filter (see **Figure 1**). *Synechococcus* cells but not heterotrophic contaminants contain chlorophyll, Chl *a*, a pigment that is excited by the SYTO 17-A laser on the FACSAria II flow cytometer. Therefore, plots of forward scatter (a measure of cell size) versus autofluorescence excited by the SYTO 17-A laser equipped with a 650–670 nm emission filter, and plots of forward scatter versus side-scatter (cell complexity) were analyzed to distinguish heterotrophic cells from the *Synechococcus* cells (see Supplementary Figure S1).

### Post-Experiment Validation of *psaA* Ecotype

When cultures reached exponential growth phase (cell density between 2 and 5 × 10^7^ cells/mL), cells were harvested by centrifugation for 30 min at 1000 × *g*. The cell pellets were frozen in liquid nitrogen and stored in a -80°C freezer. DNA was extracted and a segment of *psaA* was amplified by PCR and sequenced, as described above, to verify that the *psaA* genotype had not changed during the experiment due to the selection of a rare *Synechococcus* variant with a different light adaptation. No evidence of changes in genotypes were found in this study.

### Cell Morphology

To observe the cell morphologies of the heterotrophic contaminants and to identify any possible morphological differences among the *Synechococcus* strains, differential interference contrast and fluorescence microscopy were performed using a Nikon Eclipse 80i microscope with a Nikon Intensilight C-HGFl UV lamp and a Nikon DS-Ri1 camera. Near the end of exponential growth phase (cell density of 2 to 5 × 10^7^ cells/mL) for the 52°C pre-growth experiment, photomicrographs of cells grown under low light (25 μmol photons m^-2^sec^-1^) or high light (600 μmol photons m^-2^sec^-1^) were obtained (see Supplementary Figure S2). Composite images were then produced by combining cells (at an equal proportion) that were grown under the two light conditions into a single sample. The purpose of obtaining these images was to seek evidence of morphological acclimation to light. These same samples were also analyzed on the BD-FACSCanto flow cytometer. Cell size (forward scatter) and autofluorescence intensity were measured and compared to the microscopic observations.

### Measurement of Pigments

Total pigments were extracted from cells grown under different light conditions with 100% methanol to determine the Chl *a* and carotenoid contents. Spectroscopic measurements were carried out with a Genesys^TM^ 10 UV/Vis scanning spectrophotometer (Thermo Scientific, Rochester, NY, USA). The concentrations of Chl *a* and carotenoids were determined on the basis of equivalent cell concentrations as determined by equal OD_730__nm_ values as described in Shen et al. (2002). Phycobiliprotein (PBP) contents were determined by comparing the absorbance difference between untreated cells and cells that were incubated at 75°C for 5 min. The PBP content was estimated by the previously described procedure (Zhao et al., 2001).

### Low-Temperature Fluorescence Emission Spectroscopy

Low-temperature (77 K) fluorescence emission spectra were measured for cells grown at different light conditions using an SLM8000-based spectrofluorometer modified for computerized, solid-state operation by On-Line Instrument Systems, Inc. (Bogart, GA, USA) as described previously (Shen et al., 2008). After determination of the cell density at 730 nm, cells were adjusted to OD_730__nm_ = 0.5 in 50 mM HEPES, pH = 7 containing 60% v/v glycerol. After loading into measuring tubes, samples were incubated for 5 min in the dark and then quickly frozen in liquid nitrogen. The excitation wavelength was set at 440 nm to excite proteins containing Chl *a* selectively or to 590 nm to excite PBP selectively.

## Results

### *Synechococcus* Isolation from Mat Samples

Direct microscopic counts of mat samples from 63°C revealed that there were ∼1.8 × 10^8^ *Synechococcus* cells within the 1 mm-thick green layer sampled with a #4 cork borer, which corresponded to an approximate volume of 0.05 mL (hence ∼3.6 × 10^9^ cells/mL). After homogenization and plating on DHAY medium, growth of *Synechococcus* colonies was observed as early as day three on low-dilution plates, and as late as day 21 on high-dilution plates. The plated dilutions resulted in growth of *Synechococcus* colonies out to the 10^7^-fold dilution for the 60°C sample, and out to the 10^8^-fold dilution for the 63 and 65°C samples. The cell counts reported here were nearly identical to those reported by Brock (1978), who found the cell densities to be constant at several temperatures between 57.2 and 68°C in the Mushroom Spring mat. Thus, if this is generally true, we estimate that less than 1% of the inoculated cells from 60°C, ∼5 to 20% from 63°C, and ∼50 to 100% from 65°C formed colonies on the plated dilution series. The estimated numbers of cells in the inocula of the final dilutions demonstrating growth on plates were ∼300 from the 60°C sample, ∼30 from 63°C sample, and ∼3 from the 65°C sample. Additional extincting dilutions resulted in improvements in the efficiency of clonal isolation for all strains; specifically, ∼10 to 100% of the estimated cells in the inoculum formed colonies on plates.

Hundreds of well-isolated colonies containing cells with the typical unicellular morphology of *Synechococcus* were picked from high-dilution plates, suspended in 2 mL of liquid medium DHAY, and then gradually scaled up to larger volumes in preparation for PE classification and physiological characterization.

### Molecular and Morphological Descriptions of Strains

As shown in **Figure 1A**, microscopic observation revealed that strains contained rod-shaped, red-autofluorescing *Synechococcus* cells that were ∼8–10 μm in length. The strains also contained smaller rod-shaped cells approximately 2–5 μm in length and filamentous cells greater than 50 μm in length (**Figure 1B**). 16S rRNA pyrosequencing analyses (Supplementary Table S1) suggested that *Meiothermus* sp. and *Caldilinea aerophila*-like sequences were present in these strains, and the presence of rod-shaped and filamentous cells is consistent with morphologies of cultivated strains of *Meiothermus* sp. (Nobre and Da Costa, 2001) and *C. aerophila* (Sekiguchi et al., 2003), respectively.

Sanger and pyrosequencing revealed strains that were dominated by *psaA* sequences representative of predominant A-like PEs that are differently distributed in the vertical profile at 60 to 63°C (PEs A1, A4, and A14). These strains were assigned the names 65AY6Li, 65AY6A5, and 60AY4M2, respectively, but for simplicity they will be referred to by the *psaA*-based PEs that they represent, i.e., PEs A1, A4, and A14 (**Table 1**). Pyrosequencing analyses also revealed that *Synechococcus* strain JA-3-3Ab previously cultivated by Allewalt et al. (2006) was heavily dominated by the expected DV of PE A1 and associated random singleton variants; a single sequence corresponding to PE A14 was detected (representing less than 0.1% of the variants in the culture). In contrast, previously cultivated *Synechococcus* strain JA-2-3B′a (2–13) contained multiple high-frequency sequences representative of three different *Synechococcus* PEs (B′19, B′24, and B′11), two of which were present in substantial amounts (see **Table 1**). Similar observations were made regarding the presence of multiple *Synechococcus* PEs in strain CIW-10 (Kilian et al., 2007). In fact, the pyrosequencing data revealed that strain CIW-10 was dominated by PE B′24, which was detected in the parent culture (JA-2-3B′a (2–13)) but was not the dominant PE in that culture. A previously unidentified genotype (€ in **Table 1**) apparently arose during cultivation as the predominant variant detected in this culture.

### Adaptive and Acclimative Light Behavior

#### Effect of Heterotrophic Growth on *Synechococcus* Light Responses

**FIGURE 2:**
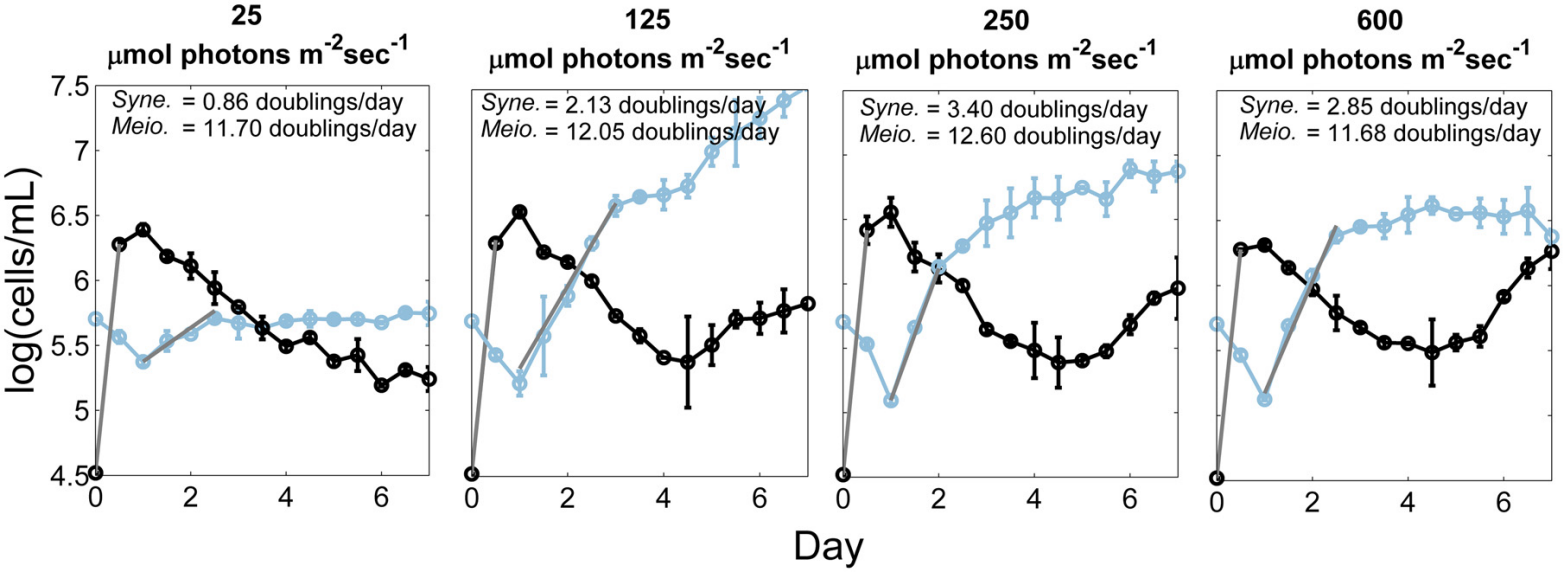
**Growth of heterotrophic and *Synechococcus* cells at different irradiances.** Number of cells of the heterotrophic population (black) and the PE A1 *Synechococcus* strain (65AY6Li; blue) at four irradiance values (25, 125, 250, and 600 μmol photons m^-2^sec^-1^), over a 7-day period at 52°C, and without any additional dissolved inorganic carbon provided to medium DHAY. The culture used as inoculum was grown at 50 μmol photons m^-2^sec^-1^. The gray lines are the least-square best fit lines that were used to calculate the growth rate during exponential growth phase. Each point is the average of two measurements and the range is shown by the bars.

A preliminary experiment was conducted to investigate the possibility that the measured light responses of the *Synechococcus* strains might be secondary to effects of light on the heterotrophic contaminants. By taking advantage of the unique fluorescence and light-scattering characteristics of the cells in these populations, the growth of both was tracked simultaneously. The results for the PE A1 strain are shown in **Figure 2**. During the first 12 h, the growth rate of the heterotrophic population (black) was nearly identical at all light intensities (∼12–13 doublings/day). During this time the *Synechococcus* population (blue) remained in lag phase. After 24 h the heterotrophic population began to decrease and the *Synechococcus* population began to increase, eventually overtaking the culture. As expected for phototrophic organisms, the growth rate of the *Synechococcus* cells varied with light intensity, whereas the growth rate of the heterotrophic cells did not. Thus, we hypothesize that the heterotrophs pre-condition the medium and that any possible impact that the heterotroph metabolic activity has on cyanobacterial response to light would be constant over the different experiments (see **Figure 2**).

#### Growth Behavior of *Synechococcus* Strains in Response to Irradiance

**FIGURE 3:**
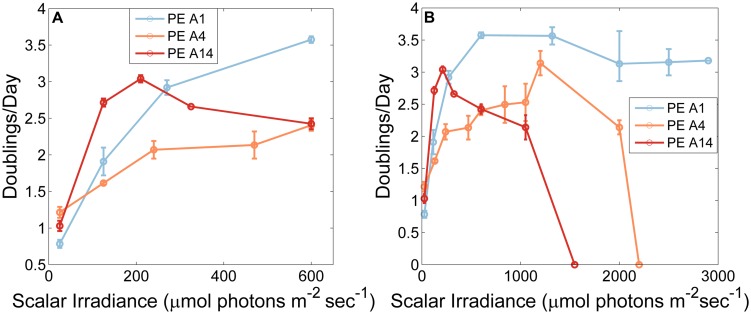
**Growth rates of *Synechococcus* strains representative of predominant PEs as a function of irradiance when grown at 60°C and sparged with 6% (v/v) CO_2_in air.** Strains used in this experiment were pre-grown at 50 μmol photons m^-2^sec^-1^, 52°C and without CO_2_ sparging **(A)** Low irradiances only. **(B)** All tested irradiances. Each point is the average of two measurements and the range is shown by the bars.

The adaptations to irradiance of the three *Synechococcus* strains grown at 60°C, when cells were pre-grown at 50 μmol photons m^-2^sec^-1^ without CO_2_ sparging, are shown in **Figure 3**. At the lowest irradiance (25 μmol photons m^-2^sec^-1^), the PE A4 and PE A14 strains grew faster than the PE A1 strain (*p* < 0.05 in a two-factor ANOVA analysis (**Figure 3A**)). The PE A14 strain grew faster than the other strains at irradiances of up to ∼200 μmol photons m^-2^sec^-1^, and the PE A1 strain grew faster than the other strains at irradiances greater than ∼250 μmol photons m^-2^sec^-1^. The highest scalar irradiance supporting growth for the PE A1 strain (**Figure 3B**, blue) was at least 2900 μmol photons m^-2^sec^-1^ at 60°C, the maximum intensity that could be achieved in the lab. The PE A4 strain (**Figure 3B**, orange) was able to grow at 2200 μmol photons m^-2^sec^-1^, but not at 2500 μmol photons m^-2^sec^-1^. The PE A14 strain (**Figure 3B**, red) had the lowest light tolerance of the three strains tested and was able to grow at 1050 μmol photons m^-2^sec^-1^, but growth was not observed at 1550 μmol photons m^-2^sec^-1^.

**FIGURE 4:**
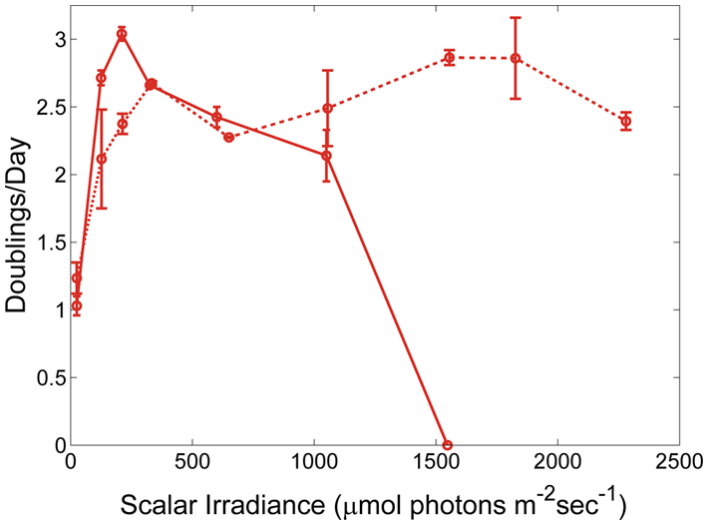
**Growth of *Synechococcus* strain representative of PE A14 when pre-grown under different conditions.** The inoculating cells were pre-grown either at 50 μmol photons m^-2^sec^-1^, 52°C and without CO_2_ sparging (solid line) or at 500 μmol photons m^-2^sec^-1^, 60°C, and with 6% CO_2_ bubbled in air (dashed line). Range bars are shown.

The differences in the observed upper-light limits of the three strains might be due to cells of different PEs having a higher or lower survival probability when shifted from very low light, 52°C and CO_2_-limiting conditions to very high light, 60°C, and CO_2_-replete conditions. When temperature- and CO_2_-shifts were eliminated and the light-shift was reduced by pre-growing at a light intensity of 500 μmol photons m^-2^sec^-1^ the PE A14 strain was able to grow at light intensities up to at least 2250 μmol photons m^-2^sec^-1^ (**Figure 4**). At light intensities ≤325 μmol photons m^-2^sec^-1^, cells pre-grown at the higher irradiance value grew after a longer lag phase than cells pre-grown at the lower light intensity, whereas at light intensities of 600 and 1050 μmol photons m^-2^sec^-1^, the opposite growth pattern was observed (Supplementary Figure S3).

#### Pigment Content of Cells Grown Under Different Irradiances

As shown in **Table 2**, the cellular contents of Chl *a*, carotenoids, and PBP were compared in cells grown at low (25 μmol photons m^-2^sec^-1^) and high (600 μmol photons m^-2^sec^-1^) irradiance. In general, Chl *a* and PBP levels were lower and carotenoid contents were higher when cells were grown at higher irradiance. However, the reductions of Chl *a* (about 45–50%) and increases in carotenoid content (about 45%) were more pronounced in the PE A4 and A14 strains compared to the PE A1 strain (about 35 and 10%, respectively). The reduction in PBP contents when cells were grown at high irradiance were of similar magnitude, but the levels were slightly lower for the strains representative of PEs A4 and A14 than for the PE A1 strain. These results indicate that all three strains regulate their pigment content as a function of irradiance in a manner similar to other cyanobacteria (Bernstein et al., 2014), but that the PE A4 and A14 strains differ in their specific responses from that of the PE A1 strain.

**Table 2:** Content of chlorophyll (Chl) *a*, carotenoids, and phycobiliproteins (PBP) in cells grown under two irradiance levels.

Strain	Irradiance^a^	Chl *a*^b^	Carotenoids^b^	Relative PBP
65AY6Li (PE A1)	25 600	6.88 ± 0.31 4.21 ± 0.26	2.29 ± 0.14 2.56 ± 0.11	135% 100%
65AY6A5 (PE A4)	25 600	7.08 ± 0.37 3.23 ± 0.19	2.31 ± 0.08 3.46 ± 0.32	147% 100%
60AY4M2 (PE A14)	25 600	7.61 ± 0.22 3.84 ± 0.17	2.25 ± 0.17 3.47 ± 0.28	145% 100%

*^a^Irradiance under which strain was grown. Units are μmol photons m^-2^sec^-1^.*

*^b^Units are μg/OD_730_/mL. OD_730_ is an abbreviation for optical density at 730 nm.*

#### Low-Temperature Fluorescence Emission Spectra

**FIGURE 5:**
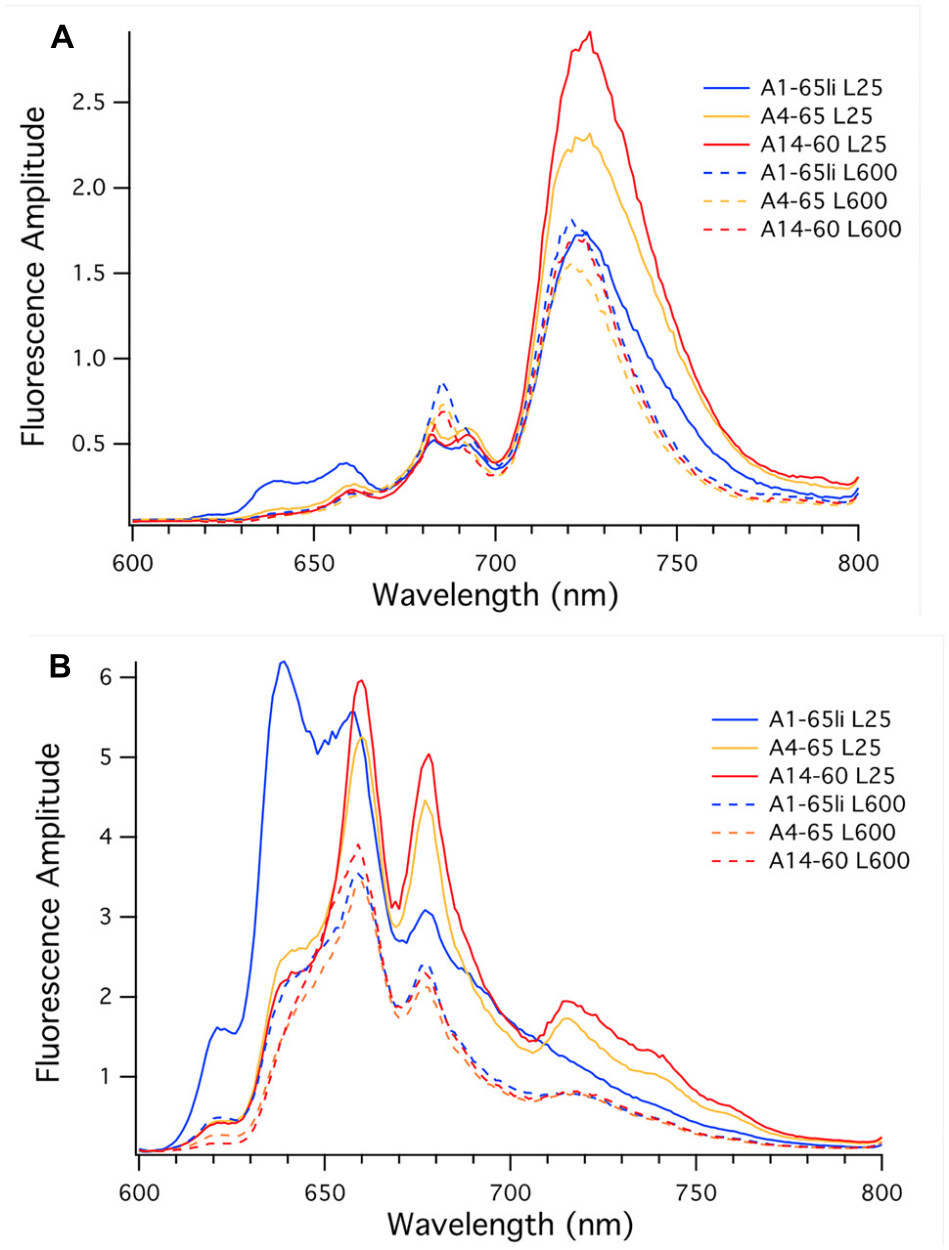
**Low-temperature fluorescence emission spectra of whole cells of *Synechococcus* strains representative of PEs A1 (blue), A4 (orange), and A14 (red). (A)** The excitation wavelength was set at 440 nm to excite chlorophyll *a* selectively. **(B)** The excitation wavelength was set at 590 nm to excite phycobiliproteins selectively. Cells were grown at 25 μmol photons m^-2^sec^-1^ (solid lines) or 600 μmol photons m^-2^sec^-1^ (dashed lines).

**Figure 5A** shows low-temperature fluorescence emission spectra for whole cells of the three strains when the excitation wavelength was 440 nm, which selectively excites Chl *a*. When cells were grown at high irradiance (**Figure 5A**, dashed lines), the overall fluorescence amplitudes for the three strains were similar but generally reflected the differences in Chl *a* content shown in **Table 2**. As judged by the similar fluorescence emission amplitudes at 683 and 695 nm, the PS II contents of the three strains were very similar, and the emission maximum (722 nm) and amplitudes for PS I emission were also similar. These data indicate that the high-irradiance adapted strain of PE A1 synthesizes slightly more Chl *a* and produces the most photosynthetic apparatus when grown under high irradiance, but that the PS I to PS II ratio of the three strains is similar when cells are grown at 600 μmol photons m^-2^sec^-1^.

**Figure 5A** (solid lines) also shows results for the three strains when cells were grown at 25 μmol photons m^-2^sec^-1^. As reflected by the similar fluorescence emission amplitudes at 683 and 695 nm, low-light-grown cells of the three strains had very similar PS II contents, but the fluorescence emission from PS I varied strikingly. The emission maximum for the strain of PE A1 had the lowest emission amplitude for PS I; the PE A4 strain had much more PS I than the PE A1 strain, and the PE A14 strain had the most PS I when cells were grown at low light. These values correlate well with the Chl *a* contents of the cells, which increased in the order PE A1 < PE A4 < PE A14. The larger PS I to PS II ratio in strains of PEs A4 and A14 and the increased total Chl *a* content are expected because PS I contains 96 Chl *a* molecules per monomer while PS II only contains 35 Chl *a* molecules per monomer (Jordan et al., 2001; Umena et al., 2011). The observation that the two low-irradiance strains have substantially broader emission bands and greater emission amplitudes between 700 and 800 nm when cells are grown under low irradiance reflects acclimative differences from the PE A1 strain. These differences could originate from Chl-protein complexes that are missing or minimally expressed in the PE A1 strain.

**Figure 5B** shows the low-temperature fluorescence emission spectra for whole cells of the three strains when the excitation wavelength is 590 nm, which selectively excites PBP. Based upon the genome sequences of *Synechococcus* strains representative of 16S rRNA genotypes A and B (Bhaya et al., 2007), these cells should synthesize only two major PBP, phycocyanin, and allophycocyanin. Consistent with the lower relative PBP contents of cells grown at high irradiance (**Table 2**), the emission amplitudes from phycocyanin (∼640 nm) and allophycocyanin (∼660 nm) and the terminal emitters of phycobilisomes (∼678 nm) were much lower in cells grown at high irradiance (**Figure 5B**, dashed lines), but the emission amplitudes were nearly identical for the three strains. These data indicate that the PBP contents of the three strains are very similar when the cells are grown under high irradiance, and that high-light grown cells have lower PBP contents than the cells that are grown under low irradiance.

Compared to the fluorescence emission spectra for strains of PEs A4 and A14, the spectrum for the PE A1 strain was quite different when cells were grown at low irradiance. The PE A1 strain had large emission peaks at 639 and 660 nm, which indicates the presence of substantial pools of uncoupled phycocyanin and allophycocyanin, respectively. The emission amplitude for phycobilisomes was much lower and there was minimal and smoothly declining fluorescence emission beyond 700 nm. The fluorescence emission spectra of cells of strains of PEs A4 and A14 grown at low light were similar and showed strong emission from allophycocyanin (661 nm) and phycobilisomes (678 nm), and intriguingly showed enhanced emission peaks at approximately 720, 740, and 760 nm. The enhanced emission at ∼720 nm might reflect greater coupling of PBP/phycobilisomes with Photosystem I in the cells of strains of PEs A4 and A14 grown at low-irradiance, which is known to occur under state transition conditions (Liu et al., 2013). However, the latter two emission bands are not observed in cells of these strains grown under high irradiance and are not observed in the PE A1 strains under either condition, and thus these must arise from an acclimative response that specifically occurs in the two low-irradiance strains. Because these emission bands are much stronger when the excitation wavelength was 590 nm, it is likely that these emission bands are associated with far-red-absorbing PBP that are present in cells of PEs A4 and A14 grown under low irradiance but that do not occur in the PE A1 strain. Thus, the fluorescence emission and pigment composition data strongly indicate that the three strains differ in both adaptive and acclimative responses. In particular, the fluorescence data clearly show that strains of PEs A4 and A14 can perform an acclimative response to growth under low-irradiance conditions that the strain of PE A1 cannot perform, which reflects an adaptive difference between PE A1 and PEs A4 and A14. This correlates very well with gene content differences in these three strains, which will be described and discussed in the third paper of this series (Olsen et al., 2015).

#### Cell Differences Observed with Microscopy and Flow Cytometry

**FIGURE 6:**
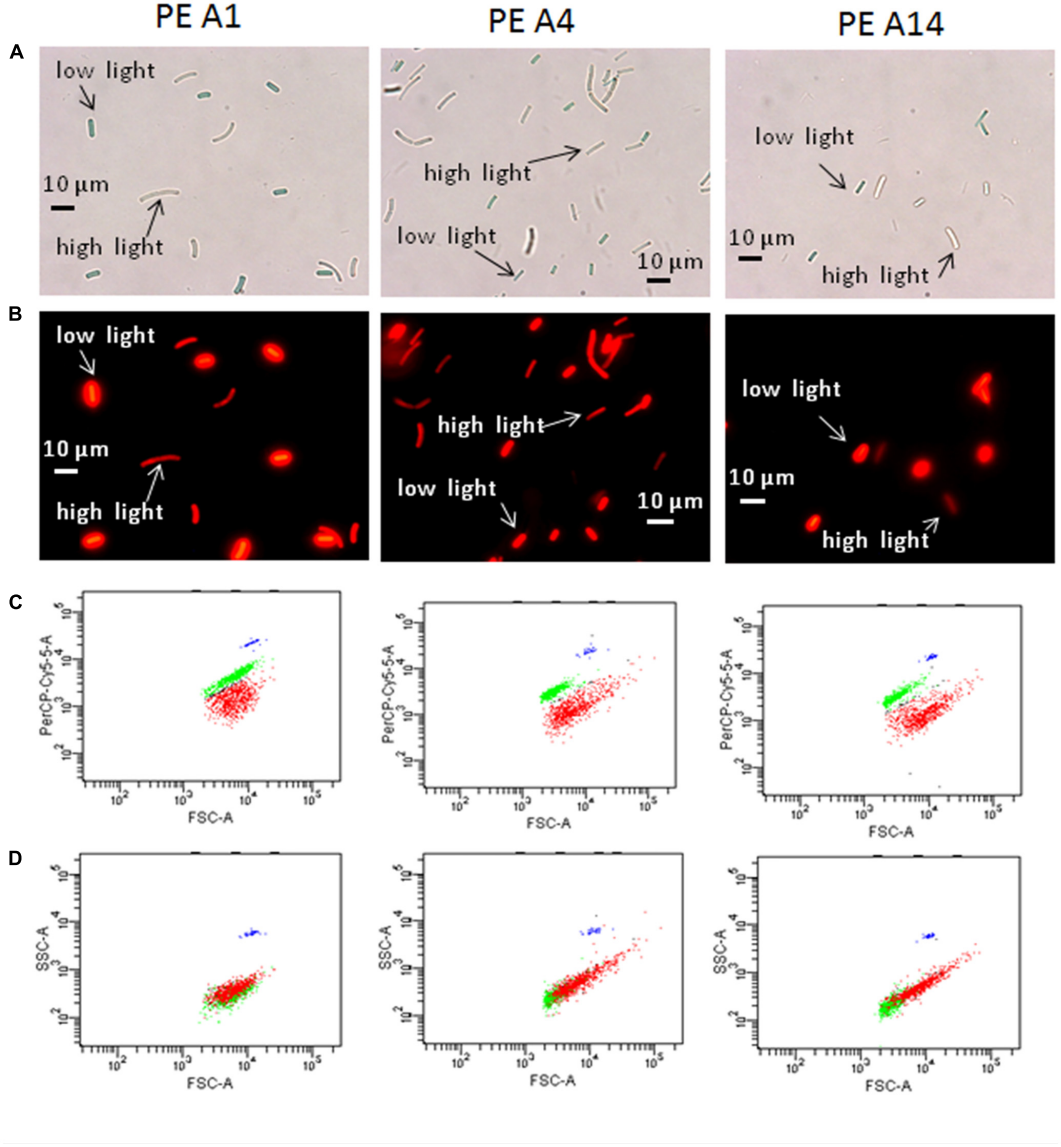
**Microscopic and flow cytometric analyses of the strains representative of PEs A1, A4, and A14 grown at 60°C and bubbled with 6% CO_2_ in air, under a low-light (25 μmol photons m^-2^sec^-1^) and a high-light condition (600 μmol photons m^-2^sec^-1^).** Pre-growth light intensity was 50 μmol photons m^-2^sec^-1^. **(A)** Differential interference contrast microscopy of low-light and high-light grown cells that were collected at the end of exponential growth phase and then mixed for comparative analyses. Scale bar is 10 μm. **(B)** Same images using fluorescence microscopy. **(C)** Scatter plots from BD-FACSCanto flow cytometer of forward scatter (cell size, horizontal axis) versus fluorescence signal (PerCP-Cy5-5-A, vertical axis) of mixed low-light and high-light samples shown in **(A)** and **(B)**. Cells grown under low irradiance are represented by the green data points and cells grown under high irradiance are represented by the red data points. **(D)** Plots of forward scatter (cell size, horizontal axis) versus side scatter (complexity, vertical axis) of mixed low-light and high-light samples shown in **(A)** and **(B)**. The blue data points represent the fluorescent counting beads that serve as a fluorescence control.

Evidence of *Synechococcus* cells acclimated to different light conditions was also provided by comparing photomicrographs and flow cytometry output of cells grown under low-irradiance and high-irradiance conditions (**Figure 6**). Cells pre-grown at 50 μmol photons m^-2^sec^-1^ and 52°C without sparging with CO_2_ were subsequently grown at 25 or 600 μmol photons m^-2^sec^-1^, 60°C and continuous sparging with 6% (v/v) CO_2_ in air. As shown in **Figures 6A,B**, cells were smaller, greener, and more brightly autofluorescent when grown at low irradiance compared to when they were grown under high irradiance. This was also observed in the flow cytometer output (**Figure 6C**), in which the cells had a higher autofluorescence signal (PerCP-Cy5-5-A) when grown under low irradiance (green data points) than when grown under high irradiance (red data points). Cells of strains representative of PEs A4 and A14 also showed a rightward shift in forward scatter and an upward shift in side scatter when grown at high irradiance (**Figure 6D**), which suggests that these cells were larger than those grown at low light. Microscopy also revealed that a large percentage of PE A4 and A14 cells (roughly 10 and 25%, respectively), but not the PE A1 cells (<1%), appeared to be unable to immediately separate from the parent cell after division (see **Figure 1C**) when grown at high light. These observations are all consistent with the data on pigment content (**Table 2**) and the fluorescence spectra (**Figure 5**) presented above.

## Discussion

The results shown here establish that *Synechococcus* strains corresponding to PEs predicted from pyrosequencing distribution analyses of *psaA* sequences exhibit different light adaptation and acclimation patterns as hypothesized by Becraft et al. (2015) in the first paper of this series. Strains of PEs A4 and A14, which predominate in the deepest part of the 1 mm-thick upper green layer of the mat, are able to grow faster at low irradiance levels typically found at this depth than a strain of PE A1, which resides above them and receives more light (see Becraft et al., 2015, Figures 3 and 4). Compared to the PE A1 strain, cells of strains of PEs A4 and A14 had higher Chl *a*, PS I, and PBP contents when grown under low irradiance. These differences in pigment content, as well as changes in the photosynthetic apparatus, are consistent with shifts in absorbance with depth that have been measured using optical microsensors (see Becraft et al., 2015, Figure 4). The low-light adapted strains also appear to be more sensitive to large changes in irradiance (combined with changes in temperature and CO_2_ supply). When light was the only parameter changed between pre-growth and experiment and the degree of increase in light was reduced, the PE A14 strain still grew faster than a strain representative of PE A1 at low irradiances, but was also able to grow at higher irradiances. This might suggest there is an evolutionary trade-off associated with the ability to grow more rapidly under low-light conditions. The two low-light adapted strains also differed from the PE A1 strain in their tendency to increase cell size and decrease post-division cell-separation rate when shifted abruptly to high irradiance conditions.

Previous studies on irradiances supporting optimal photosynthesis were conducted by observing the effects of shading native *Synechococcus* populations in Yellowstone mats with neutral-density filters (Brock and Brock, 1969; Madigan and Brock, 1977). After 10–16 days of exposure to natural light levels, and light levels reduced by 73 and 93%, samples were exposed to a range of irradiances for 5–10 min before addition of ^14^CO_2_ and incubation for 1 h. Results showed that optimal incorporation of the radiolabel occurred at lower irradiance levels in samples collected from shaded regions of the mat. The upper-light limit (or activity at the upper limit) was also reduced in samples from the shaded mat region. These results were attributed to the acclimation of a single *Synechococcus* population in the mat. Our findings and those of Becraft et al. (2015) suggest that the previously reported results were more likely due to changes in the abundances of *Synechococcus* PEs that are differently adapted to light. The decreased upper-light limit for photosynthetic fixation of ^14^CO_2_ may have occurred because of sudden exposure of the populations to light intensities much higher than those before the short-term labeling experiments were conducted.

Unfortunately, we did not recover strains representative of PE B′9, which predominates in the uppermost region of the 60–63°C mat. This may have been due to initial cultivation under low-irradiance conditions (∼50 μmol photons m^-2^sec^-1^). In a study of *Prochlorococcus* isolates, high-light adapted isolates were unable to grow when very low-irradiance conditions were provided in culture (Moore et al., 1998). Hence, we hypothesize that cells representative of the PE B′9 population may have a higher low-light threshold than the A-like strains characterized here. Interestingly, unlike *Prochlorococcus* sp. isolates, the low-light adapted *Synechococcus* PE A14 strain did have a higher upper-light tolerance when cells were not abruptly shifted to different light, temperature, and CO_2_ supply conditions. Preferential selection of A-like *Synechococcus* strains at low light might also explain differences in recovery efficiency at different temperatures, as PE B′9 is present at high relative abundance at 60–63°C, but not at higher temperatures, conditions in which A-like *Synechococcus* predominate (Becraft et al., 2015).

The results clearly demonstrate that strains previously obtained from low-dilution samples contained more than one *Synechococcus* PE. In particular, JA-2-3B′a (2–13) whose genome has been sequenced (Bhaya et al., 2007), might be a consensus genome from three or more different *Synechococcus* PEs. By screening using pyrosequencing, we were able to ensure that the strains studied here were heavily dominated by a single PE. While this approach allowed us to obtain a large number of sequences, the possibility that very rare members of other PEs are present in these strains still exists (Shrestha et al., 2013). Post-experiment sequencing confirmed that there had not been a shift in PEs under different growth conditions and that measured light relations were for a strain of a single PE. In cases not reported here, we have observed such shifts (Nowack, 2014).

The protocol we applied regularly resulted in growth of colonies from inocula that were three orders of magnitude more dilute than those used by Allewalt et al. (2006). The basis for improved recovery of *Synechococcus* from more highly diluted samples appears to be stimulation of heterotrophic contaminants by the addition of acetate and/or yeast extract. These organisms, in particular *Meiothermus*, develop rapidly in freshly inoculated strains, whereas *Synechococcus* growth initiates as these populations decline. Tank and Bryant (2015) described the purification of another strain from this mat, *Chloracidobacterium thermophilum*, from similar contaminants and the approaches they used may help us to obtain axenic *Synechococcus* cultures as well. At this time we do not know the exact cause of the sudden decline in the *Meiothermus* population. This may possibly be due to (i) nutrient depletion; the relatively small amount of organic substrate added to the medium may be depleted after 24 h of heterotrophic growth at a high rate (12–13 doublings/day), or (ii) a preference for low oxygen levels; at about the time the *Meiothermus* population reaches stationary phase, the *Synechococcus* population starts to increase exponentially, and the increased oxygen produced through photosynthesis may have an adverse effect on the heterotroph (Tank and Bryant, 2015). We are also currently investigating whether *Meiothermus* provides a nutrient(s) needed by *Synechococcus*, or alternatively, may remove toxic substances that limit the development of cyanobacteria (Sakamoto et al., 1998). Model organism experiments suggest that heterotrophs can protect cyanobacteria against reactive oxygen species as one specific beneficial interaction, but sharing of metabolites also clearly occurs (Beliaev et al., 2014).

Although we have demonstrated the existence of *Synechococcus* strains with different adaptations and acclimation responses to light, which are representative of PEs with correspondingly different vertical positioning in the upper mat green layer, it is important to keep in mind that many other physical and chemical parameters vary along the vertical aspect of the mat. For instance, when the rate of photosynthesis is high, oxygen levels are extremely high and CO_2_ is reduced to low concentrations, which can result in a sudden and significant increase in pH (Revsbech and Ward, 1984). Differences in the availability of other nutrients (e.g., nitrogen, iron, sulfate, phosphate) may also exist and change dielly. Indeed, genomic analyses of these strains by Olsen et al. (2015), as reported in the third paper in this series, are beginning to reveal such differences.

## Conflict of Interest Statement

The authors declare that the research was conducted in the absence of any commercial or financial relationships that could be construed as a potential conflict of interest.

## Footnotes

1 http://my.jgi.doe.gov/general/protocols.html

2 www.researchandtesting.com
