# Supplementary material for: The molecular dimension of microbial species: 2. Synechococcus strains representative of putative ecotypes inhabiting different depths in the Mushroom Spring microbial mat exhibit different adaptive and acclimative responses to light
Source: Front Microbiol. 2015 Jun 29;6:626. doi: 10.3389/fmicb.2015.00626 (PMC4484337; doi:10.3389/fmicb.2015.00626)
Supplement: Supplementary file 1 [file Data_Sheet_1.DOCX]

***Supplementary Material***

***Synechococcus* isolates representative of putative ecotypes inhabiting different depths in the Mushroom Spring microbial mat exhibit different adaptive and acclimative responses to light**

**Shane Nowack^1,^*, Millie T. Olsen^2^, George A. Schaible^2^, Eric D. Becraft^2^, Gaozhong Shen^3^, Isaac Klapper^1,4^, Donald A. Bryant^3,5^, and David M. Ward^2^**

^1^ Department of Mathematical Sciences, Montana State University, Bozeman, MT, USA

^2^ Department of Land Resources and Environmental Sciences, Montana State University, Bozeman, MT, USA

^3^ Department of Biochemistry and Molecular Biology, The Pennsylvania State University, University Park, PA, USA

^4^ Department of Mathematics, Temple University, Philadelphia, PA, USA

^5^ Department of Chemistry and Biochemistry, Montana State University, Bozeman, MT, USA

*** Correspondence:** Shane Nowack, University of Guelph, School of Environmental Sciences, Guelph, ON, N1G 2W1, Canada.

spnowack@gmail.com

**9. Supplementary Material**

**9.1. DNA extraction protocol for Ti454-barcode sequencing**

Extraction was performed by first adding lysozyme at 200 µg/mL (final concentration) to the sample and incubating for 45 minutes at 37ºC, mixing the sample every 15 minutes in a vortex mixer. Next, 110 µL SDS (at 1% concentration) and 200 μg/ml proteinase K (final concentration) were added to the sample and this mixture was incubated for 50 minutes at 50ºC, mixing the contents every 10 minutes. DNA was extracted by adding 950 μL of Tris-EDTA (pH 7.5) buffered phenol, mixing gently for 3 minutes, and then centrifuging for 5 minutes at 6600 × g. The aqueous layer was removed, then 450 µL phenol and 450 μL chloroform/isoamyl alcohol (24:1) were added to the sample, which was mixed gently for 3 minutes, then centrifuged for 5 minutes at 6600 × g. The aqueous layer was removed and 900 µL chloroform/isoamyl alcohol (24:1) was added and the solution was mixed gently for 3 minutes, and then centrifuged for 5 minutes at 6600 × g. The aqueous layer was removed and 40 μL 3 M NaAcetate (pH 5.2) and 1 mL 70% ethanol were added. The DNA was then allowed to precipitate overnight at -20ºC. The sample was centrifuged at 4ºC (6600 × g) for 30 minutes, washed in 0.9 mL 70% ethanol twice, and then dried using a SpeedVac (Thermo Scientific) for 10 minutes, at ambient temperature. The sample was re-suspended with 200 µL Tris-EDTA buffer (pH 7.5) and the DNA was allowed to hydrate with no mixing for two hours.

## 9.2 Supplementary Tables

**Supplementary Table 1.** Percentage of 16S rRNA Ti454-barcode variant closest relatives comprising the three *Synechococcus* isolates in this study and two of the 16S rRNA isolates from Allewalt et al. (2006).

| Closest relative^1^ | 65AY6Li  (PE A1) | 65AY6A5  (PE A4) | 60AY4M2  (PE A14) | JA-3-3Ab  (PE A1) | JA-2-3B′a (2-13)  (PE B′19/B′24) |
| --- | --- | --- | --- | --- | --- |
| *Synechococcus* spp. | 48.73 | 80.44 | 76.66 | 39.07 | 60.78 |
| *Meiothermus* spp. | 49.64 | 10.69 | 18.25 | 58.74 | 35.26 |
| *Chloroflexus* spp. | 0 | 1.97 | 0 | 0 | 0 |
| *Thermocrinis* spp. | 0 | 2.1 | 0 | 0 | 0 |
| *Caldilinea* *aerophila* | 1.22 | 2.62 | 3.01 | 0 | 0 |
| Other | .41 | 2.2 | 2.094 | 2.19 | 3.96 |

**^1^** Results provided by The Research and Testing Laboratory (Lubbock, TX), who demarcate sequences based on identity scores to well-characterized 16S sequences at the “species” level as those with greater than 97% identity (<3% divergence), and at the “genus” level as those with between 95% and 97% identity.

**9.3 Supplementary Figures**

**See 134901_Nowack_Image 1.TIF (uploaded separately)**

Supplementary Figure 1. Flow cytometer (BD FACSAria II) output of the PE A1 isolate. (A) Scatter plot showing relative fluorescence signal (SYTO 17-A) and size (FSC-A) of cells grown at 52ºC and an irradiance of 25 µmol photons m^-2^sec^-1^ in medium DHAY (without addition of any dissolved inorganic carbon) ~36 hours after inoculation. *Synechococcus* cells (green) are typically five times longer than the heterotrophic contaminants (red) that are able to pass through the 70 µm filter. *Synechococcus* cells also contain chlorophyll *a*, a pigment that is excited by the SYTO 17-A laser that the heterotrophic cells do not possess. The blue population represents the fluorescent counting beads. (B) Contour plot showing cell size (FSC-A) versus cell complexity (SSC-A). A combination of these two plots was used to differentiate heterotrophic contaminants from *Synechococcus* cells.

**See 134901_Nowack_Image 2.TIF (uploaded separately)**

**Supplementary Figure 2.** Microscopic and flow cytometric analyses of the PE A1 isolate when grown at 60ºC and bubbled with 6% CO_2_ in air, under a low-light (25 µmol photons m^-2^sec^-1^) and a high-light condition (600 µmol photons m^-2^sec^-1^).

**See 134901_Nowack_Image 3.TIF (uploaded separately)**

**Supplementary Figure 3.** Time courses for growth of the PE A14 isolate when pre-grown at 52°C and 50 µmol photons m^-2^sec^-1^ without CO_2_ sparging (solid lines) or at 60°C and 500 µmol photons m^-2^sec^-1^ with CO_2_ sparging (dashed lines). Bars are range bars.
