# Supplementary figures and images for: The molecular dimension of microbial species: 2. Synechococcus strains representative of putative ecotypes inhabiting different depths in the Mushroom Spring microbial mat exhibit different adaptive and acclimative responses to light
Source: Front Microbiol. 2015 Jun 29;6:626. doi: 10.3389/fmicb.2015.00626 (PMC4484337; doi:10.3389/fmicb.2015.00626)

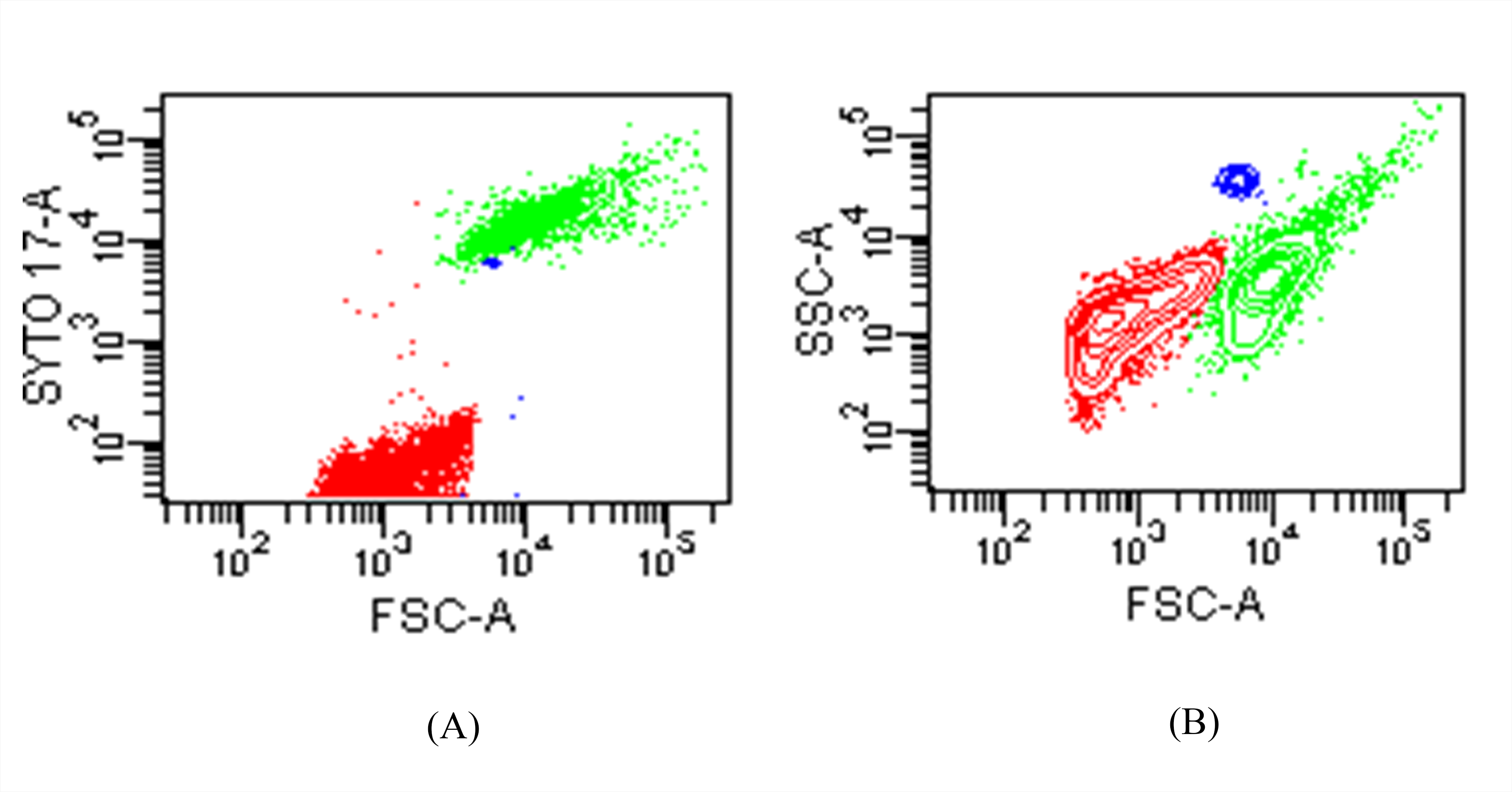

Supplement: Supplementary file 2 [file Image_1.TIF]

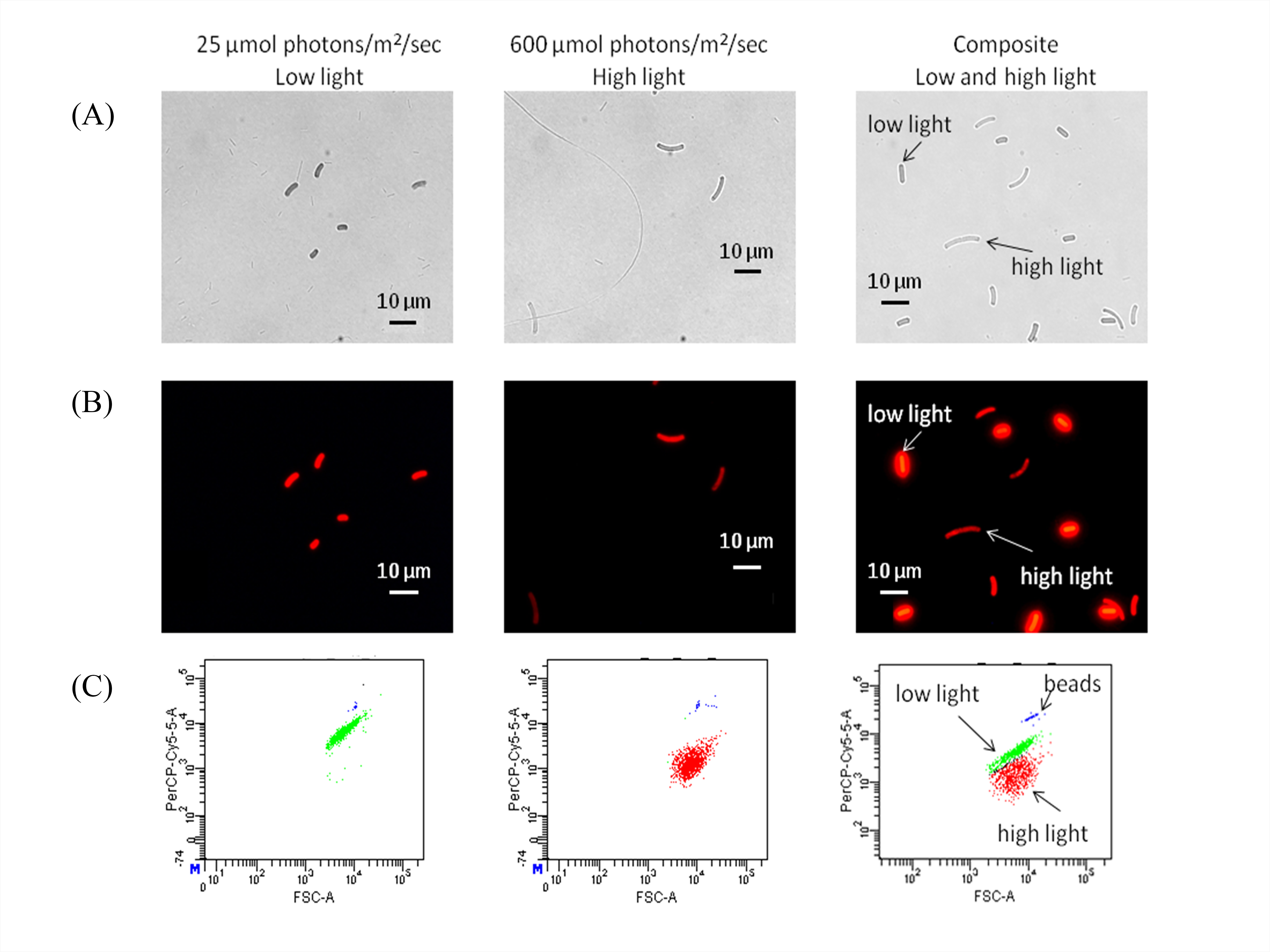

Supplement: Supplementary file 3 [file Image_2.TIF]

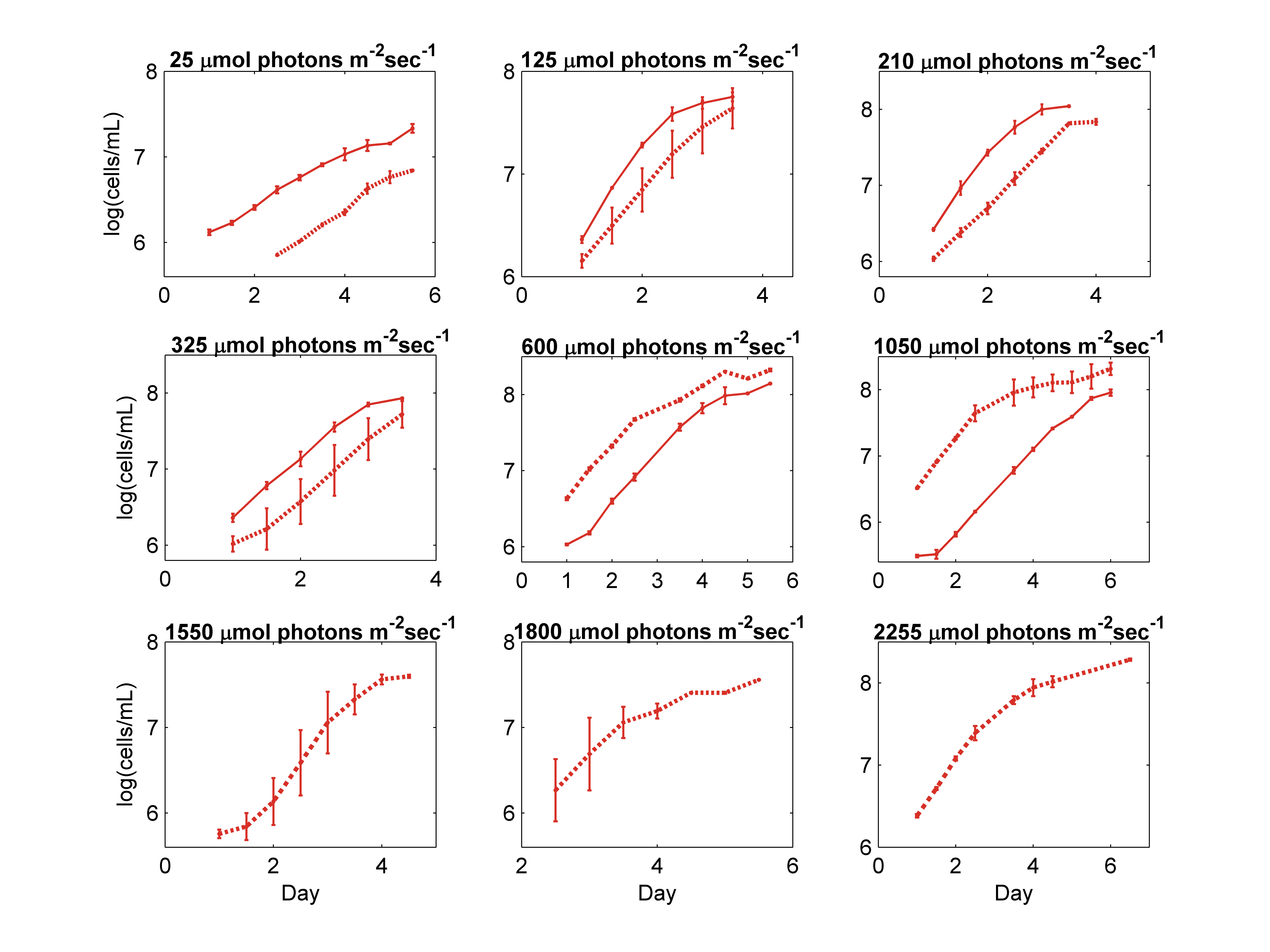

Supplement: Supplementary file 4 [file Image_3.TIF]
